# Targeting the renin-angiotensin system in sepsis-associated AKI: from pathophysiology to precision medicine

**DOI:** 10.3389/fimmu.2026.1811635

**Published:** 2026-04-15

**Authors:** Yuxin Dong, Lei Xia, Songtao Shou

**Affiliations:** Department of Emergency Medicine, Tianjin Medical University General Hospital, Tianjin, China

**Keywords:** sepsis-associated acute kidney injury, renin-angiotensin system, angiotensin II, biomarkers, precision medicine, sepsis

## Abstract

Sepsis-associated acute kidney injury (SA-AKI) is a common and life-threatening complication of sepsis. Increasing evidence suggests that dysregulation of the renin-angiotensin-aldosterone system (RAAS) is involved in its pathogenesis, but the direction of this dysregulation is not uniform. In some patients and experimental settings, elevated renin and angiotensin I levels are accompanied by an inadequate rise in circulating angiotensin II (Ang II), suggesting impaired effective Ang II generation and relative Ang II deficiency. In other contexts, persistent or excessive local Ang II signaling may continue to promote vasoconstriction, inflammation, oxidative stress, and fibrosis. These differences likely reflect the heterogeneity of sepsis across disease stages, models, biological compartments, and measurement methods. Accordingly, RAAS-targeted therapy in SA-AKI should be interpreted within a context-dependent framework: exogenous Ang II may benefit selected patients with impaired effective Ang II generation, whereas ACE2/Ang-(1-7)/Mas-based or anti-angiotensin II type 1 receptor (AT1R) strategies may be more relevant in settings of maladaptive Ang II signaling. These observations support a biomarker- and endotype-guided approach to RAAS-targeted therapy in SA-AKI.

## 1 Introduction

Sepsis-associated acute kidney injury (SA-AKI) is a frequent and severe complication in the intensive care unit (ICU), posing a significant burden on global public health (1–4). Statistics indicate that the incidence of AKI in patients with sepsis is as high as 30%–70%, and can exceed 60% in those with septic shock (1). SA-AKI also markedly increases patient mortality. Studies have shown that its in-hospital mortality rate can reach up to 25% (3). Once a patient with sepsis develops AKI, their mortality risk is 59% higher compared to patients with sepsis alone or those with AKI from other causes (e.g. nephrotoxic drugs, dehydration), and it severely impacts long-term prognosis (2, 4). Approximately 30% of SA-AKI survivors may progress to chronic kidney disease (CKD), increasing the long-term risk of end-stage renal disease and cardiovascular complications (1, 5, 6).

For a long time, the pathophysiology of SA-AKI was interpreted mainly within a traditional ischemic framework, in which sepsis-induced systemic hypotension was viewed as the primary cause of renal hypoperfusion and injury. However, this concept has been increasingly challenged. Experimental and clinical studies have shown that renal blood flow in SA-AKI is not always reduced and may even be preserved or increased in some settings (7–9). These observations, together with other evidence of microcirculatory dysfunction, inflammatory and immune-mediated injury, and cellular metabolic stress, support the view that SA-AKI is a multifactorial syndrome rather than a simple consequence of global ischemia.

In the complex pathophysiological network of SA-AKI, the renin-angiotensin-aldosterone system (RAAS) occupies an important but context-dependent position. Under physiological conditions, the RAAS is a key regulator of blood pressure, vascular tone, and sodium-water homeostasis. In sepsis, however, this system may become dysregulated in a heterogeneous manner. Current evidence suggests that RAAS dysregulation in sepsis is more complex than a simple uniform increase or decrease in angiotensin II (Ang II). In some patients and experimental settings, RAAS activation is accompanied by elevated renin and angiotensin I (Ang I) levels but an inadequate rise in circulating Ang II, indicating impaired effective Ang II generation and relative Ang II deficiency (8). This mechanism may contribute to impaired vascular tone, reduced effective renal perfusion, and hemodynamic instability. However, this pattern is unlikely to be universal. Other studies support the persistence of excessive or maladaptive Ang II signaling, particularly at the tissue level, where Ang II may continue to promote vasoconstriction, inflammation, oxidative stress, and fibrosis. These apparently divergent findings likely reflect the marked heterogeneity of sepsis, including differences in disease stage, experimental model, sampling compartment, and assay methodology. Accordingly, RAAS dysfunction in SA-AKI is best understood as a dynamic and context-dependent imbalance rather than a single fixed state.

The novelty of this review lies not merely in summarizing available RAAS-targeted therapies, but in placing these interventions within a broader framework of immune dysregulation, microcirculatory dysfunction, and kidney–organ crosstalk in SA-AKI. We further emphasize that RAAS imbalance in sepsis is heterogeneous rather than uniform, and discuss how biomarker- and endotype-guided strategies may help move the field beyond a one-size-fits-all therapeutic model. Against this background, the following sections examine the pathophysiological basis, biomarker-defined heterogeneity, and therapeutic implications of RAAS dysregulation in SA-AKI.

## 2 The pathophysiological context of RAAS dysregulation in SA-AKI

**Figure 1:**
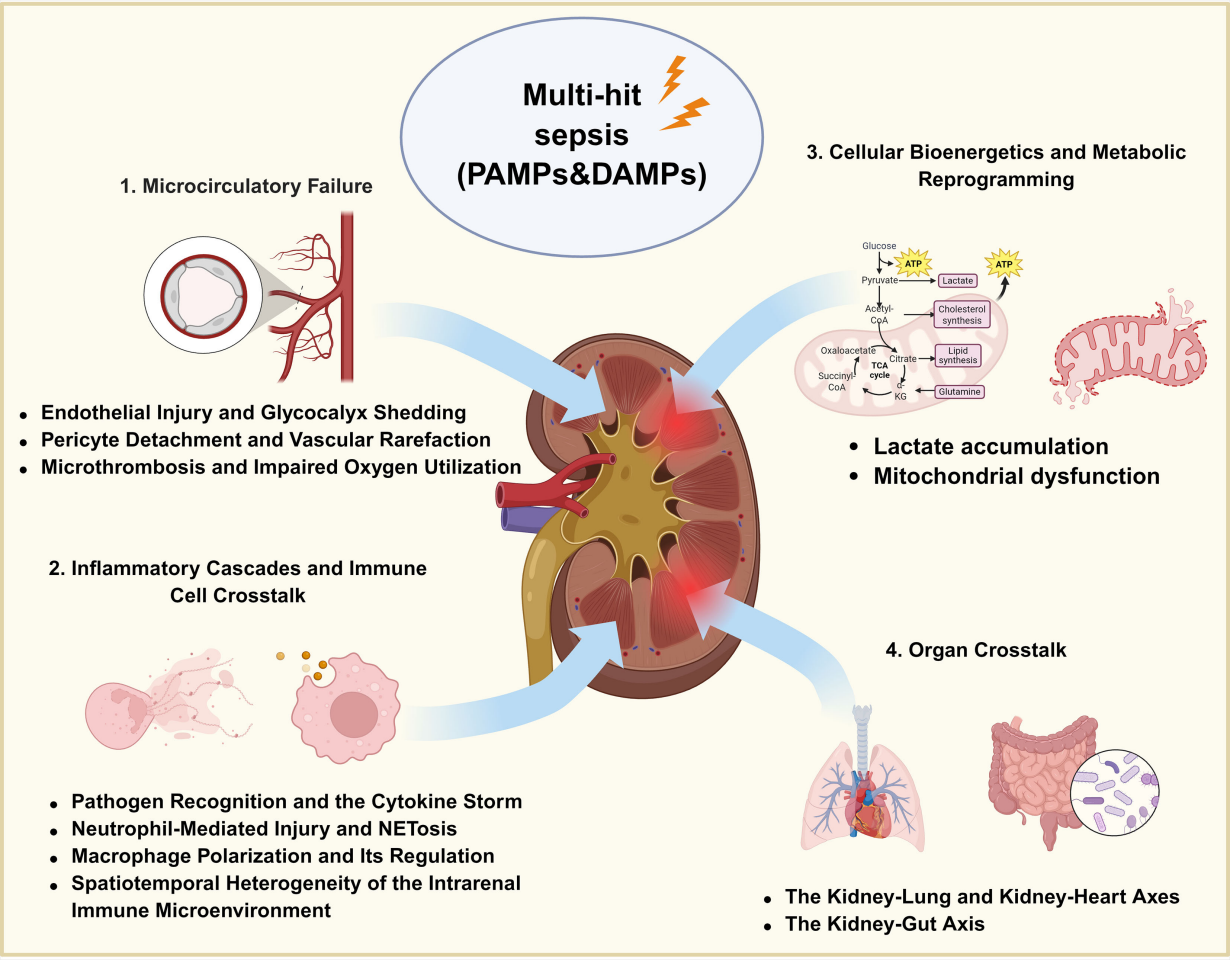
Pathophysiological context of RAAS dysregulation in SA-AKI. SA-AKI is driven by interconnected microcirculatory dysfunction, inflammatory injury, metabolic stress, and inter-organ crosstalk. Endothelial activation, glycocalyx shedding, capillary flow disturbances, pericyte dysfunction, and microthrombus formation impair renal perfusion and oxygen utilization, while inflammatory activation and mitochondrial injury further aggravate tubular damage. Persistent stress and maladaptive repair may promote fibrosis, AKI-to-CKD transition, and multi-organ dysfunction. These processes provide the pathological basis for RAAS dysregulation in SA-AKI.

SA-AKI is now recognized as a multifactorial syndrome rather than a simple consequence of global renal hypoperfusion. Its pathogenesis involves the interplay of microcirculatory dysfunction, inflammatory and immune activation, cellular metabolic derangement, and inter-organ crosstalk, ultimately leading to renal parenchymal injury, functional decline, and an increased risk of maladaptive repair and CKD progression (10) (**Figure 1**).

### 2.1 Hemodynamic and microcirculatory dysfunction

A defining feature of SA-AKI is the dissociation between systemic hemodynamics and effective renal microvascular perfusion. Even when macrocirculatory parameters such as blood pressure appear stabilized, profound perfusion abnormalities may persist within the renal microcirculation, contributing to tissue hypoxia and kidney injury (7, 9, 11). Endothelial activation and glycocalyx degradation are central to this process, as glycocalyx shedding increases vascular permeability, promotes interstitial edema, exposes adhesion molecules, and facilitates leukocyte adhesion and microthrombus formation (12–14). In parallel, pericyte contraction and detachment further impair capillary integrity and may also initiate profibrotic remodeling through pericyte-to-myofibroblast transition, thereby linking acute microvascular injury to subsequent CKD progression (15–17). These abnormalities provide the pathophysiological background in which RAAS dysregulation becomes particularly important, since relative Ang II deficiency may further impair efferent arteriolar tone, aggravate perfusion mismatch, and contribute to medullary hypoxia (11).

### 2.2 Inflammatory cascades and immune injury

Inflammation is another core driver of SA-AKI. During sepsis, pathogen-associated and damage-associated molecular patterns activate innate immune pathways, leading to cytokine release, endothelial injury, oxidative stress, and amplification of intrarenal inflammation (18–22). Neutrophils contribute not only to host defense but also to collateral tissue damage through excessive activation, reactive oxygen species release, protease secretion, and neutrophil extracellular trap formation, which further promote endothelial dysfunction and microvascular thrombosis (23, 24). Macrophage polarization also plays a dynamic role, with pro-inflammatory M1-type responses predominating in the early phase of injury, while dysregulated resolution and maladaptive immune activation may sustain tissue damage (25, 26). In addition, recent studies have highlighted marked spatiotemporal heterogeneity in the renal immune microenvironment during SA-AKI, emphasizing that inflammatory injury is compartment-specific and evolves over time (21, 22, 27, 28). Because the RAAS modulates vascular tone, endothelial biology, and inflammatory signaling, its dysregulation should be interpreted within this broader inflammatory-hemodynamic network.

### 2.3 Cellular bioenergetics and metabolic stress

Renal tubular epithelial cells are highly energy dependent and therefore particularly vulnerable in sepsis. A growing body of evidence indicates that SA-AKI is accompanied by profound metabolic reprogramming, including a shift from oxidative phosphorylation toward glycolytic metabolism, which may initially be adaptive but ultimately contributes to bioenergetic failure when sustained (29–33). At the same time, mitochondrial injury—characterized by cristae disruption, swelling, membrane potential loss, ATP depletion, and excessive reactive oxygen species generation—further compromises tubular cell survival and stress tolerance (34, 35). Multi-omics studies have also identified early metabolic perturbations in SA-AKI, supporting the concept that disordered cellular energetics is a central component of the disease process, although these biomarkers are not the focus of the present review (36). Within this metabolic context, RAAS dysregulation may further amplify hypoxic stress, endothelial dysfunction, and maladaptive cellular responses.

### 2.4 Maladaptive repair and organ crosstalk

Beyond the acute phase, unresolved inflammation, endothelial dysfunction, persistent microvascular rarefaction, and profibrotic cellular transitions may drive maladaptive repair and promote the transition from AKI to CKD (16, 17). Meanwhile, SA-AKI is not an isolated renal event but part of a broader network of inter-organ communication. Kidney injury can aggravate distant organ dysfunction through inflammatory mediators, uremic toxins, hemodynamic instability, and immune-metabolic interactions, particularly along the kidney-lung, kidney-heart, and kidney-gut axes (37–46). These systemic interactions reinforce the concept that SA-AKI is a dynamic and multisystem disorder. Against this background, the key issue is not simply whether the RAAS is activated in sepsis, but how its activation becomes dysregulated and no longer translates into effective hemodynamic and tissue-level regulation, thereby contributing to hemodynamic instability, inflammatory amplification, and kidney injury.

## 3 RAAS imbalance in SA-AKI: classical and counter-regulatory axes

**Figure 2:**
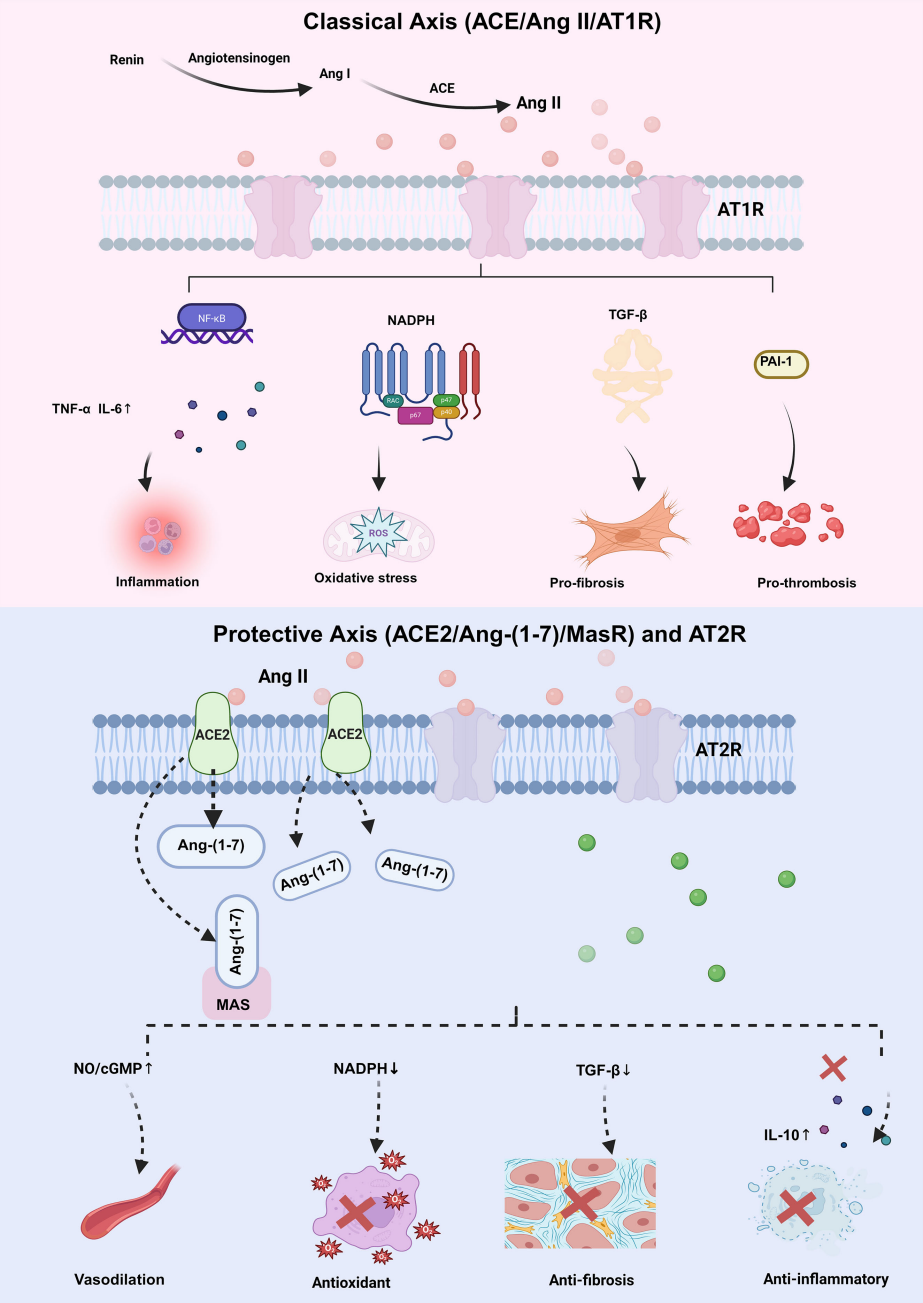
Imbalance of the RAAS in SA-AKI. Under physiological conditions, a balance is maintained between the classical and counter-regulatory RAAS axes. In sepsis, this equilibrium is disrupted. The classical axis is activated, with elevated renin and angiotensin I but inadequate effective Ang II generation, contributing to relative Ang II insufficiency. At the same time, endothelial injury may promote ACE2 shedding and alter the function of the protective axis. Ang-(1-7), acting through MasR, exerts anti-inflammatory, antioxidant, and vasodilatory effects. Together, these changes illustrate the heterogeneous and compartment-dependent dysregulation of the RAAS in SA-AKI.

The RAAS is not a simple linear system but a complex, dynamic network composed of multiple axes with distinct and even opposing functions. In SA-AKI, the balance between the classical injury-promoting RAAS axis and the counter-regulatory protective axes is profoundly disturbed, leading to the dominance of damaging effects (47, 48) (**Figure 2**). Rather than representing a single uniform state, RAAS dysfunction in SA-AKI likely reflects a dynamic imbalance between systemic hemodynamic insufficiency and persistent local maladaptive signaling. The major classical and counter-regulatory axes of the RAAS, including their principal enzymes, peptides, receptors, and biological effects, are summarized in **Table 1**.

**Table 1:** Major classical and counter-regulatory RAAS axes in SA-AKI.

Feature	Classical axis (ACE/Ang II/AT1R)	Protective axis (ACE2/Ang-(1-7)/MasR)	Protective axis (AT2R)
Key Effector Peptide	Ang II	Ang-(1-7)	Ang II
Key Receptor	AT1 Receptor (AT1R)	MasR	AT2 Receptor (AT2R)
Vascular Effects	Potent vasoconstriction, increased permeability	Vasodilation, decreased permeability	Vasodilation (via kinin/NO system)
Inflammatory Effects	Pro-inflammatory: ↑ TNF-α, IL-6; ↑ NF-κB activation; leukocyte recruitment	Anti-inflammatory: ↓ cytokine release, ↓ leukocyte infiltration	Anti-inflammatory: ↑ IL-10, promotes M2 macrophage polarization
Coagulation Effects	Pro-thrombotic: ↑ platelet aggregation, ↑ PAI-1	Anti-thrombotic	Likely anti-thrombotic
Oxidative Stress	Pro-oxidant: ↑ ROS production (via NADPH oxidase)	Anti-oxidant	Anti-oxidant
Tissue Remodeling/Fibrosis	Pro-fibrotic: ↑ TGF-β, ↑ collagen deposition	Anti-fibrotic: ↓ TGF-β, ↓ extracellular matrix production	Anti-fibrotic, anti-proliferative
Overall Role in SA-AKI	Pathogenic: Drives microvascular failure, inflammation, and maladaptive repair.	Protective: Counter-regulates the classical axis, promotes resolution of inflammation.	Protective: Promotes adaptive immune responses and tissue repair.

↑, increase/upregulation; ↓, decrease/downregulation.

### 3.1 The classical axis: a relative Ang II deficiency

In sepsis, the classical RAAS axis is often strongly activated but may become functionally dysregulated rather than uniformly overactive (49). Elevated renin levels are thought to reflect not only early renal hypoperfusion, but also loss of negative feedback when effective circulating Ang II generation is inadequate. In parallel, widespread endothelial injury may impair angiotensin-converting enzyme (ACE) activity, contributing to a mismatch between RAAS activation and effective Ang II generation. This pattern is characterized by markedly increased renin and Ang I levels but an insufficient rise in Ang II, resulting in a high Ang I/Ang II ratio (50). In addition, dipeptidyl peptidase 3 (DPP3), which is released into the circulation during cellular injury, may further contribute to impaired effective Ang II activity, suggesting that it functions not only as a biomarker of severe cellular injury but also as a pathophysiological mediator (49, 51).

At the same time, the consequences of Ang II dysregulation in sepsis are compartment- and stage-dependent. It is therefore important to distinguish between the relative inadequacy of circulating Ang II in the acute hemodynamic phase and the persistent local tissue-level effects of Ang II signaling. Even when systemic Ang II activity is functionally insufficient, Ang II that is generated locally may still exert potent pathological effects through the angiotensin II type 1 receptor (AT1R) (52). Through AT1R, Ang II promotes the release of pro-inflammatory cytokines such as TNF-α and IL-6, upregulates endothelial adhesion molecules including ICAM-1, activates NF-κB signaling, enhances oxidative stress through NADPH oxidase activation, and contributes to microvascular thrombosis and tissue injury (53–56). In later phases, sustained Ang II/AT1R signaling may further promote fibrotic remodeling and contribute to the transition from AKI to CKD (57).

The relevance of AT1R-mediated signaling in sepsis is also supported by experimental and translational studies. Early work demonstrated that AT1R-dependent pathways contribute to hemodynamic responses in sepsis models, highlighting the importance of classical RAAS signaling during septic shock (58). More recently, the role of Ang II–AT1R signaling in the pathophysiology of vasodilatory shock and RAAS dysfunction in sepsis was further emphasized (59). Taken together, these findings suggest that AT1R remains an important component of septic pathophysiology, although its net effects may vary according to disease stage, biological compartment, and the balance between systemic and local RAAS activity.

Accordingly, the central issue in acute septic shock is not simply Ang II excess or deficiency in absolute terms, but a context-dependent imbalance: inadequate effective circulating Ang II may compromise vascular tone and glomerular filtration, whereas persistent local Ang II/AT1R signaling may continue to drive inflammation, oxidative stress, and fibrotic injury. This duality suggests that the classical RAAS axis in SA-AKI cannot be interpreted using a single uniform model.

### 3.2 The counter-regulatory RAAS axes in sepsis

The RAAS not only contains classical damaging pathways but also possesses protective counter-regulatory pathways capable of opposing these effects. The functional status of these pathways collectively determines the ultimate fate of the kidneys and other tissues.

It should be emphasized that the direction of Ang II dysregulation in sepsis is not uniform across studies. Clinical investigations showing elevated renin levels, altered Ang I/Ang II balance, or favorable responses to exogenous Ang II support the hypothesis that some patients with vasodilatory septic shock have impaired effective circulating Ang II generation (49, 50, 60, 61). In contrast, experimental and translational studies indicate that Ang II may remain pathogenic at the tissue level, particularly through AT1R-mediated inflammatory, oxidative, and profibrotic signaling (52–57, 59). These discrepancies likely reflect differences in disease stage, biological compartment, and measurement methodology.

#### 3.2.1 The ACE2/Ang-(1-7)/MasR axis

The ACE2/Ang-(1-7)/MasR axis is a major counter-regulatory component of the RAAS. ACE2 degrades Ang II into the heptapeptide Ang-(1-7) (62). By binding to the Mas receptor, Ang-(1-7) exerts effects that generally oppose those of the Ang II/AT1R axis, including vasodilatory, anti-inflammatory, anti-oxidative, and anti-fibrotic actions (63–66).

Some experimental and clinical studies have reported increased circulating ACE2 activity and alterations in the Ang-(1-7)/Ang II balance in sepsis or septic shock, findings that may reflect altered processing of Ang II and changes in the balance between classical and counter-regulatory RAAS peptides in the circulation (50, 67). At first glance, these observations appear to suggest activation of the counter-regulatory axis. However, they do not necessarily indicate preservation of its protective function at the tissue level.

Indeed, other studies have shown that elevated circulating ACE2 (cACE2) is associated with worse clinical outcomes in sepsis. Increased cACE2 levels have been associated with ICU mortality and a higher risk of AKI in septic patients (68). Similarly, increased cACE2 activity has been reported in patients with septic shock (67). These findings suggest that higher circulating ACE2 does not simply reflect enhanced tissue protection. Rather, it may result from cleavage and shedding of ACE2 from injured endothelial surfaces into the bloodstream. In this context, elevated cACE2 may serve as a marker of endothelial damage and loss of tissue-level ACE2 activity, rather than a sign of preserved protective signaling (67, 68).

Taken together, these observations indicate that impairment of the protective RAAS axis in sepsis cannot be understood simply as reduced or increased activity in absolute terms. Instead, it likely reflects a compartment-dependent process in which circulating ACE2-related activity may increase, while tissue-level ACE2 function is disrupted or lost. This distinction may help explain why the protective axis appears activated in some measurements, yet ineffective in preventing ongoing inflammation, endothelial injury, and organ dysfunction.

#### 3.2.2 The angiotensin II type 2 receptor

Although most of the detrimental effects of Ang II are mediated by the AT1R, its binding to the AT2R typically activates protective signaling pathways that generally oppose or counterbalance AT1R-mediated injury signaling (69–71). AT2R activation has anti-inflammatory, tissue-reparative, and renoprotective functions (69, 72). A key role is its regulation of immune responses, particularly macrophage polarization: AT2R activation significantly promotes the transition of macrophages from a pro-inflammatory M1 phenotype to an anti-inflammatory and reparative M2 phenotype (73, 74). This is crucial for suppressing excessive inflammation, facilitating efferocytosis, and initiating tissue repair processes.

## 4 Biomarker-guided RAAS endotyping in SA-AKI

**Figure 3:**
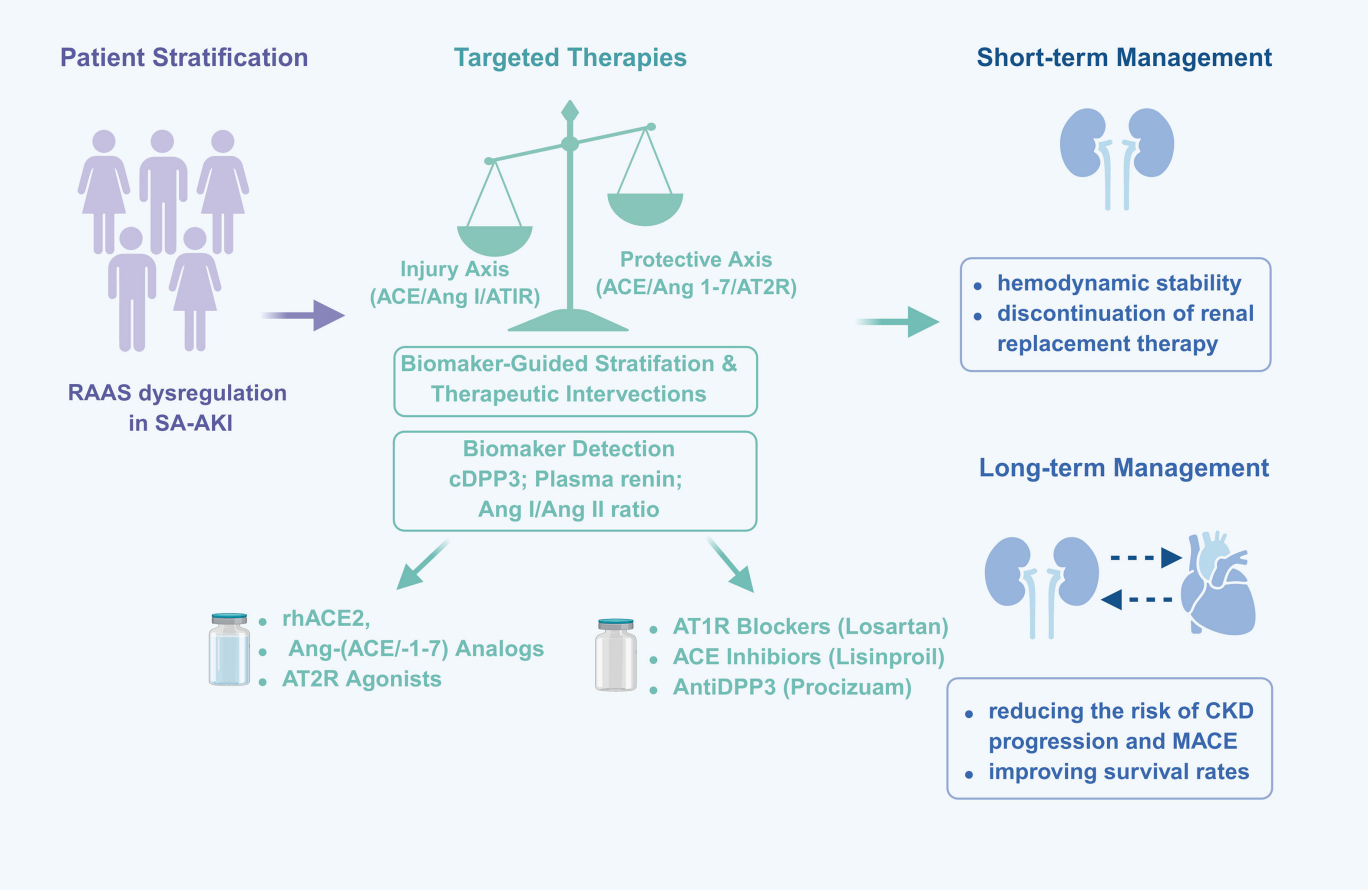
Framework for biomarker-guided RAAS-targeted interventions in SA-AKI. This figure illustrates a proposed framework for stratifying patients with septic shock into distinct pathophysiological endotypes using biomarkers. The central problem is an imbalance between the injury-promoting classical axis (ACE/Ang II/AT1R) and the counter-regulatory protective axes (ACE2/Ang-(1-7)/MasR and AT2R). Biomarkers such as plasma renin, cDPP3, and the Ang I/Ang II ratio may help identify patients with different patterns of RAAS dysregulation and guide targeted therapeutic interventions. Potential interventions include Ang II supplementation, rhACE2, Ang-(1-7) analogues, AT2R agonists, and anti-DPP3 strategies. Potential goals include short-term hemodynamic stabilization and renal recovery, as well as long-term reduction in progression from AKI to CKD and major adverse cardiovascular events.

SA-AKI exhibits a high degree of heterogeneity in clinical presentation, pathophysiology, and prognosis. This necessitates the use of biomarkers to finely stratify patient populations, identify subgroups with common molecular characteristics and pathological mechanisms, and thereby implement more targeted treatment strategies (**Figure 3**).

### 4.1 Renin: a key biomarker for assessing RAAS dysregulation and predicting therapeutic response

Plasma renin concentration is a crucial indicator of the activation state of the RAAS. Recent studies suggest that dynamic changes in renin levels may have predictive value for acute kidney injury and may outperform static measurements in some settings (75). More importantly, the biomarker subgroup analysis of the ATHOS-3 trial revealed that septic shock patients with high baseline renin levels experienced a significant reduction in mortality after receiving Ang II therapy. These findings support a biomarker-guided approach in which renin may help identify patients with RAAS dysfunction and relative Ang II deficiency who are more likely to benefit from Ang II supplementation.

### 4.2 Circulating dipeptidyl peptidase 3: a marker of myocardial depression and a modulator of the RAAS

DPP3 is an intracellular, cytosolic enzyme found in numerous cells, including endothelial cells, red blood cells, and cardiomyocytes (76). During severe sepsis and tissue injury, it is released into the bloodstream. High cDPP3 levels are thus a marker of severe cell injury and strongly predict organ failure (77, 78). Critically, cDPP3 is not just a biomarker but an active pathophysiological mediator; it accelerates the degradation of Ang II, directly contributing to a phenotype characterized by impaired effective Ang II activity. It also exerts direct myocardial depressant effects (76). Therefore, cDPP3 levels help identify a specific shock phenotype characterized by myocardial depression and vasoregulatory dysfunction. Specific antibodies against DPP3 (such as Procizumab) have been shown in preclinical studies to rapidly improve cardiac function and renal hemodynamic indicators, suggesting it may become a future therapeutic target (51, 78, 79).

### 4.3 Integration and application of biomarkers in clinical trials: the DARK-sepsis study

The DARK-Sepsis trial represents a significant exploration of the precision medicine concept in the field of sepsis. This randomized controlled trial is designed to prospectively validate the efficacy of renin and DPP3 as biomarkers to guide the selection of vasoactive drugs in patients with septic shock (80). The trial may provide an important framework for testing whether biomarker-based stratification can be translated into clinically useful vasoactive treatment strategies (**Table 2**).

**Table 2:** Emerging biomarkers of RAAS dysregulation in SA-AKI.

Biomarker	Biological function/source	Clinical relevance/Key findings
Plasma Renin	Enzyme released by juxtaglomerular cells; rate-limiting step of the RAAS. Its level reflects perceived renal hypoperfusion and effective RAAS activation.	Elevated plasma renin is strongly associated with worse outcomes in vasodilatory shock and SA-AKI and may outperform traditional markers such as lactate for mortality prediction. In addition, high baseline renin may identify patients more likely to benefit from exogenous Ang II, supporting a biomarker-guided treatment strategy.
Circulating Dipeptidyl Peptidase 3 (cDPP3)	Cytosolic peptidase released during cellular injury; degrades Ang II and other vasoactive peptides.	High cDPP3 levels are associated with severe organ dysfunction, vasopressor requirement, renal replacement therapy, and mortality. As a pathophysiological biomarker, cDPP3 may indicate both severe cellular injury and functional RAAS disruption, and experimental inhibition has shown beneficial effects on cardiac function.
Ang I/Ang II Ratio	Ratio of angiotensin I to angiotensin II; reflects ACE-dependent conversion efficiency.	A high Ang I/Ang II ratio is associated with impaired effective Ang II generation, endothelial dysfunction, and worse clinical outcomes. It may serve as a mechanistic biomarker of impaired effective Ang II generation and help identify patients with inadequate circulating Ang II activity.

## 5 From pathophysiology to clinical practice: current and emerging RAAS-targeted therapies

Translating our deep understanding of the complex role of the RAAS into effective clinical interventions is a core task of current SA-AKI research (**Table 3**).

**Table 3:** Key clinical studies of RAAS modulation in sepsis and vasodilatory shock.

Trial/Study	Design	Patient population	Intervention	Key findings & significance
ATHOS-3 (60, 92)	Phase 3 RCT	321 patients with catecholamine-resistant vasodilatory shock	Ang II vs. Placebo	Ang II improved mean arterial pressure and supported its role as an effective vasopressor in refractory vasodilatory shock.
ATHOS-3 (AKI-RRT Subgroup) (81)	*Post-hoc* analysis	105 ATHOS-3 patients with AKI requiring renal replacement therapy at baseline	Ang II vs. Placebo	Ang II was associated with improved survival and renal recovery in a high-risk subgroup, suggesting potential benefit in patients with severe SA-AKI.
ATHOS-3 (Biomarker Subgroup) (61)	*Post-hoc* analysis	ATHOS-3 patients with available biomarker samples	Ang II vs. Placebo	Elevated baseline renin identified patients more likely to benefit from Ang II, supporting a biomarker-guided treatment strategy.
RAAS Inhibitor Discontinuation Study (84)	Target trial emulation	27, 003 patients receiving chronic RAAS inhibitors who developed hospital-acquired AKI	Discontinuation vs. continuation of RAAS inhibitors within 2 days of AKI	Temporary discontinuation of RAAS inhibitors during acute illness was associated with improved short-term outcomes, supporting context-dependent RAAS management.
DARK-Sepsis (80)	Pilot RCT	Patients with vasodilatory shock receiving moderate-dose norepinephrine	Ang II vs. standard of care	This study is designed to evaluate whether baseline renin and cDPP3 can predict vasopressor response, providing a clinical framework for biomarker-guided RAAS therapy.

### 5.1 Exogenous Ang II: insights from the ATHOS-3 trial

The landmark ATHOS-3 trial demonstrated that for patients with catecholamine-resistant vasodilatory shock, exogenous Ang II effectively increases mean arterial pressure (MAP) and shows a trend toward improved survival (60). However, the most impactful findings came from *post-hoc* analyses. A *post-hoc* analysis focusing on patients with co-existing AKI revealed that the effect of Ang II treatment on 28-day survival varied with AKI severity: in the overall AKI population, there was no significant difference in mortality between the Ang II and placebo groups (53% vs. 63%); however, in patients with stage 3 AKI, the mortality rate in the Ang II group was significantly lower than in the placebo group (48% vs. 67%). Additionally, the Ang II group showed superior outcomes in terms of MAP response and days free from renal replacement therapy (81).

The rationale for Ang II as a replacement therapy is most strongly supported by a pre-specified biomarker analysis of the ATHOS-3 trial. The patients who were sensitive to the Ang II dose (dose could be down-titrated to ≤5 ng·kg^-1^·min^-1^ after 30 minutes of treatment) had better clinical outcomes, with a significantly higher 28-day survival rate compared to patients requiring higher doses (59% vs. 33%) (84). This analysis suggested that patients with high baseline renin levels (indicative of an activated, uncoupled RAAS and likely Ang II deficiency) had a significant mortality benefit from Ang II treatment, whereas those with low renin did not. These findings support the clinical relevance of renin-based stratification and suggest that RAAS-targeted therapy may be most effective when guided by biomarker-defined hemodynamic endotypes.

### 5.2 The clinical dilemma of RAAS inhibitors: a context-dependent re-evaluation

Regarding RAAS inhibitors, particularly angiotensin-converting enzyme inhibitors and angiotensin receptor blockers, the existing evidence on their use in the context of sepsis and AKI shows that their effects are highly dependent on the clinical scenario, posing a challenge for decision-making.

#### 5.2.1 Potential protective effect of pre-hospital long-term use

Multiple large-scale observational studies have indicated that patients who were on long-term ACEI or ARB therapy before the onset of sepsis have a significantly lower risk of in-hospital mortality. One possible explanation is that prior exposure to these drugs may improve tissue resilience to subsequent ischemic or infectious insults through anti-inflammatory and anti-oxidative effects (82).

However, a large, population-based cohort study reached a diametrically opposite conclusion: compared with the use of calcium channel blockers, chronic use of RAASi was associated with a higher risk of developing AKI requiring RRT and septic shock in patients with sepsis (83). A systematic review by Tibi et al. also confirmed the confounding nature of the existing evidence, noting that patients’ underlying comorbidities are a key factor influencing outcomes (47). These inconsistent findings may reflect several factors. First, results may differ depending on the comparator group, particularly when patients receiving RAAS inhibitors are compared with untreated individuals rather than with those receiving other antihypertensive agents. Second, residual confounding by indication remains difficult to exclude, since long-term RAAS inhibitor users often have more severe diabetes, chronic kidney disease, or heart failure, all of which independently increase the risk of poor outcomes in sepsis. Third, in the early phase of sepsis, RAAS inhibitors may worsen hypotension, so their acute hemodynamic effects could outweigh any potential protective benefit associated with prior exposure. Overall, the impact of pre-hospital long-term RAAS inhibitor use on outcomes in septic patients remains uncertain. It is likely to depend on individual patient characteristics and clinical context, and further well-designed prospective studies are needed.

#### 5.2.2 Benefit of discontinuation of RAAS inhibitors during AKI

In contrast to the protective effect seen with long-term pre-hospital use, continuing RAASi during AKI correlates with adverse outcomes. A large-scale target trial emulation study involving over 27, 000 patients showed that for long-term users who developed hospital-acquired AKI, promptly discontinuing RAAS inhibitors within two days of diagnosis significantly reduced 30-day all-cause mortality (discontinuation group 4.36% vs. continuation group 5.91%) and 180-day mortality risk. This suggests that in the stress state of acute kidney injury, timely interruption of RAAS inhibitor exposure is beneficial for short-term prognosis (84).

#### 5.2.3 Decision-making for restarting therapy after AKI recovery

Whether and when to restart RAAS inhibitor therapy during the AKI recovery phase is another complex clinical decision. Some studies have shown that although restarting therapy may cause a transient decrease in glomerular filtration rate, for survivors of the most severe AKI (e.g. KDIGO stage 3), continued use of these drugs is associated with lower long-term all-cause mortality (85). However, for patients without clear cardiovascular or renal disease indications, restarting therapy may increase the risk of adverse kidney events. Therefore, the decision requires a careful, individualized assessment of the potential cardiorenal protective benefits versus the risks, based on the patient’s specific indications.

### 5.3 New therapeutic frontiers: targeting protective RAAS pathways

Importantly, RAAS-targeted therapies should not be regarded as universally interchangeable, because the pattern of RAAS dysregulation in sepsis may vary across patients, disease stages, and biological compartments. Recombinant human ACE2 (rhACE2) has been proposed as a potential strategy to restore balance within the RAAS by converting Ang II into the protective peptide Ang-(1–7) (86, 87). This approach may be particularly relevant in settings characterized by persistent or maladaptive Ang II signaling, especially at the tissue level (86, 87). However, because some patients with sepsis may instead exhibit systemic RAAS uncoupling with inadequate circulating Ang II generation, the therapeutic value of rhACE2 in SA-AKI should be interpreted cautiously and may depend on patient endotype and timing of intervention. It has already demonstrated significant protective effects on the lungs and kidneys in preclinical studies related to COVID-19 (88). Together, these observations suggest that selective modulation of protective RAAS pathways may offer new therapeutic opportunities in SA-AKI, but their application will likely require a biomarker- and endotype-guided rather than one-size-fits-all approach.

## 6 Long-term consequences of sepsis: from AKI to chronic disease

The impact of SA-AKI extends far beyond the acute hospitalization phase. For survivors, this acute event often leads to long-term changes in renal function and structure, significantly increasing the future risk of developing CKD (89). Persistent local activation of the classical Ang II/AT1R axis within the kidney microenvironment is considered a core driver of this progression. This is distinct from the systemic RAAS uncoupling and relative Ang II deficiency seen in the acute shock phase. In the chronic (AKI-to-CKD) phase, local Ang II drives maladaptive repair.

### 6.1 Maladaptive repair and the AKI-to-CKD transition

After severe AKI, renal repair is often incomplete and may be accompanied by persistent low-grade inflammation, microvascular rarefaction, and progressive fibrosis, thereby promoting the development of CKD. Studies have shown that the local RAAS in the kidney remains continuously activated long after the acute phase, with upregulated Ang II/AT1R axis signaling driving this vicious cycle by promoting inflammatory responses, oxidative stress, and pro-fibrotic processes (90).

### 6.2 Post-AKI cardiovascular risk: the link between RAAS and MACE

SA-AKI survivors may face an increased long-term risk of major adverse cardiovascular events (MACE) (1, 5, 6). The sympathetic nervous system and RAAS, activated during the acute phase, may remain in a hyperactive state long after the injury, promoting myocardial hypertrophy, vascular stiffness, and cardiac remodeling. Additionally, volume overload due to impaired renal function, accumulation of uremic toxins, and a chronic inflammatory state are closely associated with atherosclerosis and worsening cardiac function, with the RAAS likely contributing to several of these interconnected pathological processes (91).

### 6.3 Clinical management implications for SA-AKI survivors

Given that persistent RAAS activation simultaneously drives both CKD progression and cardiovascular risk, restarting or continuing RAAS inhibitors (such as ACEI/ARB) in SA-AKI survivors is theoretically protective but requires a highly individualized risk assessment. A target trial emulation study suggested that, among prior users who developed hospital-acquired AKI, discontinuing RAAS inhibitors within two days was associated with lower 30-day and 180-day mortality (84). Therefore, clinical decisions must comprehensively consider the patient’s renal function recovery, proteinuria levels, hemodynamic status, and comorbidities. Current evidence supports the establishment of specialized follow-up clinics for AKI survivors to implement prudent RAAS inhibitor management strategies through regular monitoring of renal function, urinary protein, and cardiovascular indicators, thereby reducing the risk of long-term complications.

## 7 Future perspectives

Future research and clinical management of SA-AKI will likely move toward a more precise and individualized approach. Rather than relying on a one-size-fits-all model, treatment strategies may increasingly be guided by differences in underlying pathophysiology. With the integration of multi-omics data, clinical biomarkers, and computational approaches, it may become possible to better characterize heterogeneous patterns of RAAS dysregulation and identify clinically relevant RAAS endotypes that can inform therapeutic decision-making. At the same time, therapeutic development is likely to focus on more selective interventions that promote intrarenal repair while minimizing adverse systemic hemodynamic effects. Applying such targeted strategies during the acute or subacute phase may help improve renal recovery, reduce progression to CKD, and lower the long-term risk of cardiovascular complications.

## 8 Conclusion

In conclusion, SA-AKI is a multifactorial syndrome in which immune dysregulation is a central driver of endothelial injury, microcirculatory failure, and kidney dysfunction. RAAS abnormalities should therefore be interpreted within this broader immunological and vascular context rather than as isolated endocrine disturbances. Because RAAS imbalance in sepsis is heterogeneous and context-dependent, its therapeutic modulation is unlikely to follow a one-size-fits-all model. The added value of this review lies in linking RAAS-targeted interventions to a broader pathophysiological and precision medicine framework that integrates immunology, organ crosstalk, and biomarker-guided stratification.
